# How do manipulation checks interfere with the inference of causal relationships?

**DOI:** 10.3758/s13428-024-02573-3

**Published:** 2024-12-30

**Authors:** Yuhwa Han, Wooyeol Lee

**Affiliations:** Department of Psychology, Chungbuk National University, 1 Chungdae-Ro, Seowon-Gu, Cheongju, Chungbuk 28644 Republic of Korea

**Keywords:** Manipulation check, Mediation analysis, Manipulation intensity, Measurement error, Multicollinearity

## Abstract

This study investigates the performance of mediation analyses, including manipulation check variables, in experimental studies where manipulated psychological attributes are independent variables. We simulated the level of manipulation intensities and measurement errors of the manipulation check variable to test the validity of the analytic practice. Our results showed that when manipulation is successful and measurement error is low, mediation analyses with the manipulation check variable revealed an unstable path coefficient and standard error. Moreover, many of the detected indirect effects were inconsistent mediation situations. However, when individual differences in psychological attributes remained within the condition (low manipulation intensity) and the manipulation check variable contained low measurement error, the indirect effect indicated the validity of the manipulation. We discuss the implications of our findings for the use of manipulation checks in experimental research.

In the field of psychology, experimental studies that manipulate psychological attributes, such as self-esteem or emotions, are often conducted (e.g., Chinchilla et al., 2022; Kaluza et al., 2022; Lench & Bench, 2015; Lench et al., 2016; Vázquez et al., 2021; Zhao & Biernat, 2022) to infer the attributes’ causal relationships with dependent variables. As psychological attributes are inherently continuous, experimental studies often involve categorizing them through manipulation. In this study, we refer to such experimental designs as experiments manipulating psychological attributes (EMPs). This contrasts with experiments in which the researcher manipulates physical attributes. For example, in the Stroop task (Stroop, 1935), the researcher manipulates the “color” of the experimental stimulus. In this task, the attributes that the researcher hypothesizes will affect the dependent variable are directly manipulated.

EMPs are more susceptible to the threat of internal validity than typical experiments. The validity of the results can be affected by both “what” and “how much” something is changed through the experimental manipulation. In response, Lench et al. (2014) proposed a method to strengthen causal explanations in EMPs by including manipulation check variables as mediators (known as the total manipulation check [TMC]). Herein, we discuss the conditions under which Lench et al.’s (2014) method may be useful and demonstrate these conditions through a simulation study.

## Threats to internal validity in EMPs

We discuss two factors that impede causal inference in EMPs: manipulation intensity and construct validity.

### Manipulation intensity

Experiments that manipulate a continuous psychological attribute have underlying assumptions: The degree of a psychological characteristic is subject to change, and individuals assigned to the same experimental condition will have the same psychological state after the experimental manipulation. This is based on the assumption, in causal inference, that the cause is a given, resulting in no errors (Pearl, 2014). However, when manipulating continuous psychological attributes, this assumption is only hypothetical. Given that individuals possess distinct baselines of psychological attributes and are affected by experimental treatments in various ways, it is worth noting that experimental manipulations serve to modify rather than eradicate individual differences at the level of psychological attributes. For example, assume that the manipulation of participants’ anxiety levels involved showing an anxiety-inducing video to the experimental group and a tranquil video of landscapes to the control group. Even participants in the experimental group who watched the same video would experience different levels of anxiety. Therefore, individual differences in psychological attributes still exist after the manipulation.

**Fig. 1:**
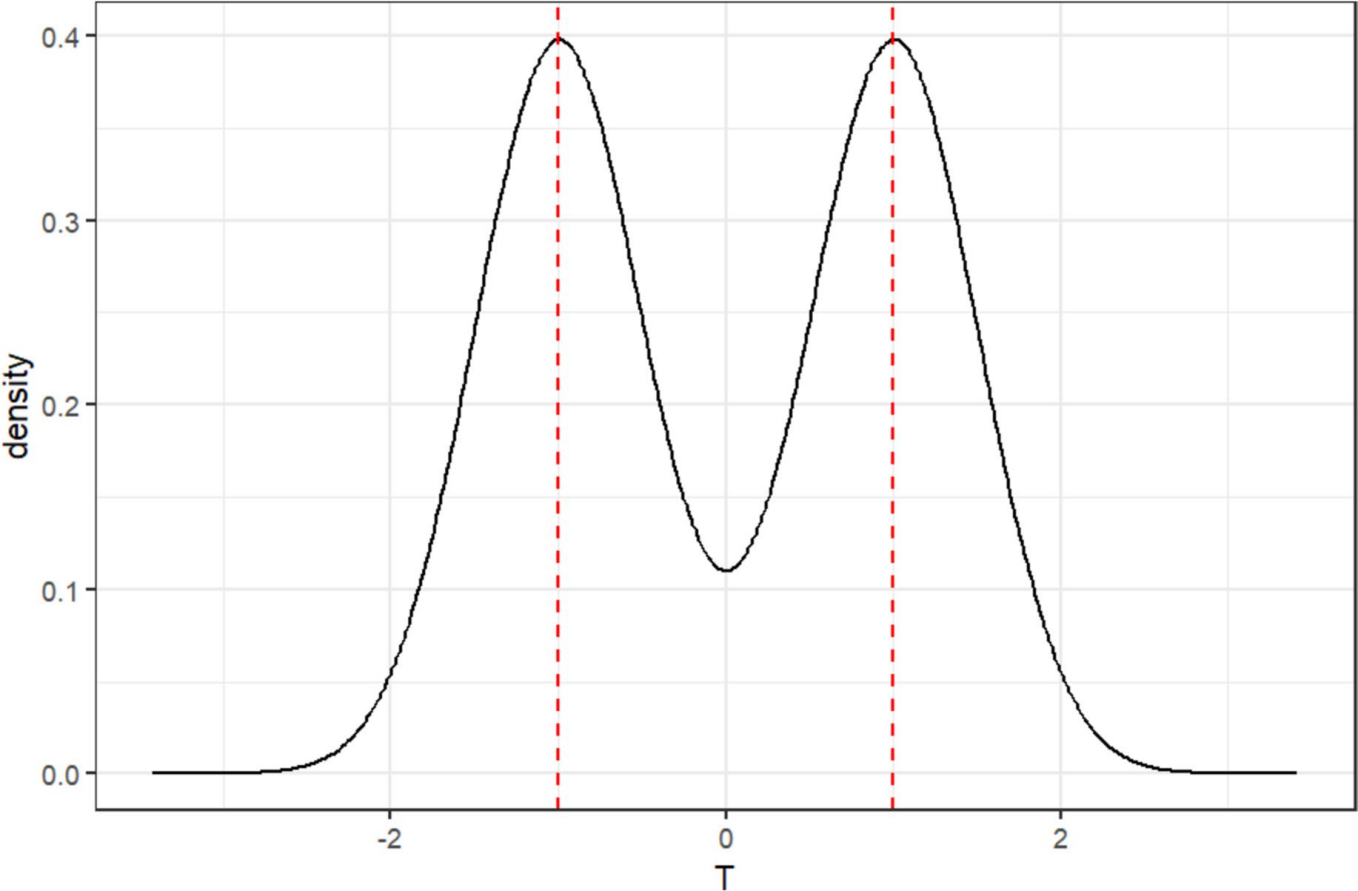
Distribution of the hypothetical psychological attribute T (the *x*-axis is an arbitrary unit)

Individual differences in psychological attributes are distributed bimodally after the manipulation (Fig. 1). The mean of the two distributions in Fig. 1 represents the average of the psychological attributes in the experimental and control groups, such as the level of the independent variable ($\overline{{T }_{t}}$ and $\overline{{T }_{c}}$, respectively). The variance of the two distributions is the individual difference (${e}_{r}$) remaining after the experimental manipulation. Assume the independent variable is *X*. The relationship among the level of *X*, the level of the individual psychological attributes after the manipulation, and the individual differences is described in Eq. 1.

1 $$X=\overline{T }=T+{e}_{r}$$

Manipulation intensity is the ratio of the mean magnitude between levels of an independent variable to the standard deviation of individual differences. Alternatively, it can be expressed as the correlation coefficient between the independent variable and the psychological attribute coded by the coding scheme. The greater the manipulation intensity, the less the overlap in psychological traits between participants assigned to each level of the experimental condition. In EMPs, the effectiveness of the manipulation is critical to the intended causal interpretation. Individual differences within experimental conditions contaminate the effect of the manipulated independent variable because statistical analyses, such as independent samples *t*-tests, do not consider these individual differences.

### Construct validity, manipulation check variables, and measurement error

Researchers assume that they are changing a psychological attribute through experimental manipulation. However, it is possible to change something unintentionally and observe a change in the dependent variable as a result. This may be another threat to internal validity. In EMPs, the manipulation check (MC) is conducted to test the effectiveness and construct validity of the manipulation. The manipulation check variable is a separate measure designed to determine whether the experimental treatment succeeded in producing the internal state that constitutes the conceptual variable (Carlsmith et al., 1976) and serves as evidence of the validity of the psychological attributes (Sigall & Mills, 1998). Therefore, the MC confirms the psychological attributes targeted by the experimental manipulation (Ejelöv & Luke, 2020) and reflects the validity of the manipulation (Shadish et al., 2002). In the previous example, the anxiety levels of both groups were measured using a reliable scale to assess the effectiveness of the video-based manipulation and compare anxiety levels between the experimental and control groups. This type of MC is based on the effectiveness of the manipulation and is standard in experimental studies manipulating psychological attributes. An MC based on manipulation validity can be conducted by verifying that the manipulation changed only anxiety and not other psychological attributes (e.g., fear).

Considering that the manipulation check variable is obtained by measuring the manipulated psychological attributes, measurement error is built into the variable. Ignoring systematic errors, all errors in the manipulation check variable are considered random errors. In this study, these random errors are referred to as measurement errors. The configuration of the manipulation check variable represented by *M* is shown in Eq. 2.

2 $$M=T+{e}_{m}$$

## Using manipulation check variable: TMC

In most cases, the validity of the manipulation is supported theoretically (Campbell & Cook, 1979) or assumed based on previous studies (Ejelöv & Luke, 2020). It is also possible to check for construct validity by conducting a pilot experiment independent of the main experiment that includes a manipulation check procedure. However, many social experiments choose to conduct a manipulation check simultaneously with the main experiment.

Lench et al. (2014) argued that the validity and effect of the manipulation could be examined simultaneously by testing the mediation effect of the manipulation check variable on the relationship between the manipulated independent variable (*X*) and the dependent variable (*Y*). Several studies have adopted the procedure, TMC (Chinchilla et al., 2022; Kaluza et al., 2022; Lench & Bench, 2015; Lench et al., 2016; Vázquez et al., 2021; Zhao & Biernat, 2022).

**Fig. 2:**
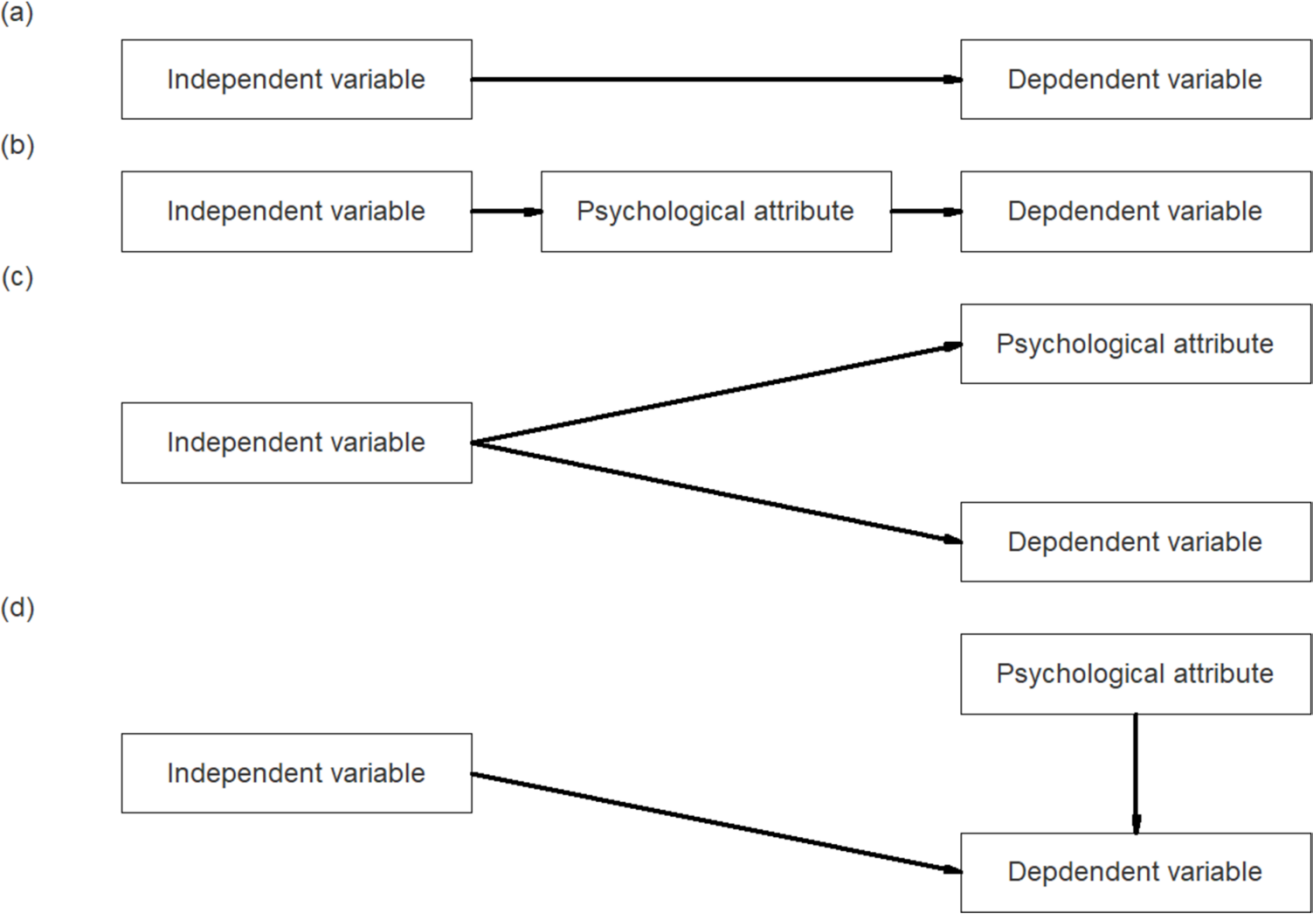
Relationships between constructs. **a** The surface relationship between the independent variable (*X*) and the dependent variable (*Y*). **b** Path diagram when the *X→Y* relationship is significant and *X* is a valid manipulation of the psychological attribute *T*. **c** Path diagram when the *X→Y* relationship is significant, but the relationship between *T* and *Y* is not true. **d** Path diagram describing another situation where the manipulation is not valid (adapted and modified from Lench et al., 2014)

The logic of the TMC procedure (Fig. 2) is as follows: In an experiment in which *X* is experimentally manipulated to test the relationship between a psychological attribute ($T$) and a $Y$, the researcher tests the relationship between $X$ and $Y$ at the operational level (Fig. 2a). However, the researcher’s hypothesis is about the relationship between $T$ and $Y$ at the conceptual level. *X* is a variable that operationally defines *T*, so researchers should test both $X\to T$ and $T\to Y$ paths (Fig. 2b).

We can consider two cases where the $X\to T\to Y$ path is not confirmed. In the first situation, the operational-level relationship exists but the conceptual-level relationship does not; that is, the relationship between $X\to T$ and $X\to Y$ is verified, but the relationship of $T\to Y$ is not. We can then infer that $X$ affects $T$ and $Y$ simultaneously (Fig. 2c). In the second situation, the relationship between $X\to T$ does not exist, but the $T\to Y$ and $X\to Y$ relationships do. In this situation, the experimental manipulation did not work as the researcher intended, but the manipulated variable and the psychological attribute independently affected the dependent variable. This situation represents a weak manipulation and is unlikely to occur in a sound experimental design (Fig. 2d).

To apply TMC, the researcher uses a mediation analysis in which the relationship between $X$ and $Y$ is mediated by the manipulation check variable $M$, which is considered a proxy for $T$. Mediation analysis tests the significance of the indirect effect (*ab*), defined as the product of the effect of $X\to M$ (*a*) and the effect of $M\to Y$ (*b*). Baron and Kenny (1986) proposed a sequential method for mediation analysis in which the total effect (*c*) is tested first, followed by the indirect effect (*ab*). On the other hand, Hayes (2022) argued that only testing *ab* can be meaningful. According to Lench et al. (2014), in TMC, it makes no sense to omit the significance test for the $X\to Y$ effect and test only the indirect effect. It is also meaningless to test for direct effects in a mediation model with the aim of distinguishing between complete and incomplete mediation. The significance of the indirect effect can be tested with a *Z*-test (Baron & Kenny, 1986) or a bootstrap test (Preacher & Hayes, 2004).

## Factors that affect TMC

The TMC process faces a paradox: the more successful the manipulation check, the less reliable the results. The independent and the manipulation check variables are conceptually the same variables with only a difference in the level of measurement. These two variables are highly related. Multicollinearity problems can easily arise when these two variables are included in a model at the same time. Multicollinearity is a correlation between two variables or a variable that is a linear combination of the values of two variables. A high correlation indicates multicollinearity between variables, but the reverse is not true (Alin, 2010). In the presence of multicollinearity, the standard error of the regression coefficient is estimated to be large, and the precision of the regression coefficient is reduced (Alin, 2010), which will ultimately affect the type I error rate and the power of the hypothesis testing. Lench et al. (2014) acknowledge TMC’s multicollinearity problem. They argued that TMC is not needed if the experimental manipulation is effective enough to leave no room for doubt about the construct validity of the independent variable. However, they have not provided specific situations in which researchers can claim that their manipulation is effective and that construct validity is not an issue.

Manipulation intensity and measurement error in the manipulation check are likely to affect TMC results. The more effective the experimental manipulation of *T* (i.e., the smaller the individual differences in *X*) and the smaller the measurement error of the manipulation check variable, the greater the multicollinearity among the variables in the model. As mentioned earlier, TMC results are expected to be unreliable in situations of high multicollinearity.

Nevertheless, large measurement errors in the manipulation check variable can also adversely affect TMC results. One of the assumptions for causal inference through mediation analysis is the absence of measurement errors in the variables included in the model (Baron & Kenny, 1986). However, several studies have demonstrated the consequences of using variables with measurement error in mediation analysis (Cohen et al., 2003; Gastonguay et al., 2023; Liu & Wang, 2021; VanderWeele et al., 2012). In general, the measurement error of $X$ in the mediation model causes the total effect of $X$ to be smaller than its true value (le Cessie et al., 2012), and the measurement error of the mediator causes the indirect effect of $X$ to be underestimated (VanderWeele et al., 2012). These findings are consistent with classical test theory, which states that measurement error has the property of attenuating correlations between variables (Spearman, 1904). Therefore, if the manipulation check variable with a significant measurement error is included in a mediation model, the validity of the model can be expected to be low.

Notably, the psychological attribute *T* is a variable that cannot be observed directly, but must be inferred indirectly from *X* and *M*. While the researcher can only observe the directly manipulated variable *X*, the measured variable *M*, and the correlation between *X* and *M*, an unobservable variable *T* can alter the relationship between *X* and *M*. Consequently, the true relationship between *T* and *X* (manipulation intensity) and the relationship between *T* and *M* (measurement error) may be different, even though the observed correlation between *X* and *M* is identical. Additionally, as described above, the validity of a mediation model, such as TMC, may vary depending on the relationship between “$T$ and $X$” and “$T$ and $M$” because the multicollinearity between variables in the mediation model may be affected. This raises the need for Monte Carlo simulation experiments, in which the researcher manipulates the parameters and investigates the results of the experiment.

## Research questions

In summary, EMPs contain factors that can threaten internal validity. In this study, we investigate the practice of including manipulation checks in the analysis of EMPs using simulation methods. The purpose of the simulation study is to investigate how causal inference using the TMC procedure is affected by the manipulation intensity of the independent variables and the measurement error of the manipulation check variable.

## Simulation study

This study used Monte Carlo simulations to investigate the impact on research results of two issues that may arise in experimental situations in which psychological attributes are manipulated. The first issue is the effect of individual differences on research results (reflecting the manipulation intensity) that are ignored in manipulating and analyzing psychological attributes. The second issue revolves around the ramifications of introducing the manipulation check variable of the manipulated psychological attributes into the model to test research hypotheses concerning experimental condition effects. Specifically, we set the degree of manipulation of the independent variable (manipulation intensity, *rTX*), the measurement error of the manipulation check variable (*rTM*), the relationship between the manipulation check and the dependent variables (*rMY*), and the true effect (*rTY*) as simulation conditions, and examined the performance of the mediation analysis. The R code for data generation and analysis in this study is provided in Supplementary Material S1 and is available in the corresponding author’s OSF repository (https://osf.io/hwkq3/).

### Simulation design and data generation

In this study, the independent variable (*X*) was created first, and from it, the psychological attribute (*T*), the observed manipulation check variable (*M*), and the dependent variable (*Y*) were created through mathematical transformation. *X* consisted of two levels (control and experimental), and the number of participants in each level was equal to 50. The two levels were coded as 1 and − 1 for effect coding, respectively. *T* was randomly generated from a normal mixture distribution that followed a normal distribution at each level. The standard deviation of the two distributions (individual differences) was manipulated to be equal. *M* was randomly generated from *T* by manipulating the correlation coefficient, and *Y* was generated from *T* and *M* using mediation analysis formulas. *T*, *X*, *M*, and *Y* were all fixed at a mean of 0 and a standard deviation of 1 to obtain standardized path coefficients for the mediation analysis results. The formulas used to create and transform the variables in this study are presented in Supplementary Material S2. Below, we describe each level of the manipulated variables in detail.

### Manipulation intensity (rTX)

The manipulation intensity for the psychological attributes was set at four levels, 0.5, 0.7, 0.95, and 1, in units of the correlation coefficient between *T* and *X* (*rTX*). At the *rTX* = 0.5 level, the distributions of *T* for participants assigned to the two levels of the experimental condition were *N*(− 0.5, ${0.865}^{2}$) and *N*(0.5, ${0.865}^{2}$). The manipulation intensity at this level was 1.16 in effect size (*d*) units (see Ruscio, 2008, for *r*-to-*d* conversions). At the *rTX* = 0.7 level, the distributions of *T* within the experimental condition levels were *N*(− 0.707, ${0.707}^{2}$) and *N*(0.707, ${0.707}^{2}$), respectively, and the manipulation intensity in effect size units was 2.00. At the *rXT* = 0.95 level, the distributions of *T* for each level of the experimental condition were *N*(− 0.95, ${0.3}^{2}$) and *N*(0.95, ${0.3}^{2}$), respectively, with a manipulation intensity of 6.085 in effect size units. Finally, the *rTX* = 1 level was an idealized manipulation situation with no individual differences in psychological attributes after the experimental manipulation. The two levels of *X* were coded − 1 for all participants in one level and 1 for all participants in the other level.

### Manipulation check (rTM) measurement error

The degree to which the manipulation check variable reflects the psychological attributes (measurement error) was set at 0.5, 0.7, and 0.95 in terms of the correlation coefficient between *T* and *M* (*rTM*). At each level, the variance explained by *M* is 25%, 49%, and 91% of *T*, respectively. Among them, the *rTM* = 0.95 level would reflect a situation of high multicollinearity.

### Relationship between the manipulation check and the dependent variables (rMY)

The relationship between the manipulation check and the dependent variables was set to three levels in the correlation coefficient between *M* and *Y* (*rMY*): 0, 0.3, and 0.5. A level of *rMY* = 0 indicated no relationship between the manipulation check and the dependent variables. According to Lench et al. (2014), this level refers to a situation in which a mean difference in *Y* as a function of *X* cannot be causally explained (as shown in Fig. 2c). In contrast, values of *rMY* = 0.3 and 0.5 indicate that the relationship between the manipulation check and the dependent variable exists.

### True effect (rTY)

The effect of the psychological attributes on the dependent variable was set at 0.3 and 0.5 in terms of the correlation coefficient between *T* and *Y*. The levels of *rTY* = 0.3 and 0.5 indicated that *T* affected *Y* and referred to medium and large effect sizes, respectively, according to Cohen’s (1988) guidelines.

The four manipulated simulation variables in this study were designed to be fully crossed, resulting in a total of $4\times 3\times 3\times 2=72$ simulation conditions, and the sample size (*N*) was fixed at 100. A total of 1000 replications were randomly and independently generated from the parameters of each simulation condition.

### Analytical methods

The following analyses were performed for each replication where data generation was successful. First, the mean, standard deviation, and correlation coefficient between all variables were calculated to confirm whether the data generation was successful. Second, the mean difference of *Y* according to the level of *X* was tested using a *t*-test to select experimental conditions that met the conditions proposed by Lench et al. (2014). Third, following Baron and Kenny’s (1986) method, we conducted a multiple regression analysis with *X* and *M* as predictors and *Y* as the dependent variable. Fourth, we conducted a mediation analysis including *X* and *M* to test for indirect effects. The *mediate* function of the *psych* package (R package version 2.3.3; Revelle, 2023) was used to test for indirect effects. In the mediation analysis and indirect effects test, the independent variable was set as *X*, the mediator was the manipulation check variable *M*, and the dependent variable was set as *Y*. The path coefficients *a, b, c*, *c′,* and *ab* of the mediation analysis were recorded in each analysis. The significance of the indirect effect *ab* was tested by performing 1000 additional bootstrap resamplings of the indirect effect *ab*. Whenever the zero value did not fall within the 95% bootstrap confidence interval, the indirect effect was considered significant. The level of significance was recorded as 1 for a significant effect and 0 for a nonsignificant effect.

### Evaluation measures

The main interest of this study is to examine the performance of the mediation analysis with the manipulation check variable. After conducting the above analysis using 1000 replication data obtained from each simulation condition, the results were combined, and the proportion of the number of replications meeting the following conditions was used as the detection rate for the indirect effect. The first condition is that, as the practice of Baron and Kenny (1986), (1) the *t*-test result of *X→Y*, (2) the simple regression coefficient of *X→M*, and (3) the regression coefficient of *M* in the multiple regression analysis of *X*, *M→Y *are all statistically significant. The second one is that both the *t*-test result of *X→Y* and the indirect effect *ab* by the bootstrap method are significant.

### Expected results

We expected that the manipulation intensity and measurement error manipulated in this study would affect the results of the mediation analysis including the manipulation check variable. As discussed in the introduction, a situation with high manipulation intensity and low measurement error is ideal. At the same time, this situation is also prone to multicollinearity. Multicollinearity can destabilize the estimation of regression coefficients (Cohen, 1978; Cohen et al., 2003) and lead to unsystematic changes in the indirect effect detection rate. In the present study, high indirect effect detection was expected in conditions with high manipulation intensity and low measurement error, but multicollinearity caused inconsistent detection rates. Furthermore, multicollinearity is likely to be greater for larger *rMY* and *rTY*, and the instability of the indirect effect detection rate is affected by multicollinearity.

## Results

We analyzed only 68 of the 72 simulation conditions, excluding four conditions where it was mathematically impossible to generate the observed variable *Y* from the given parameters ([*rTX, rTM, rMY, rTY*] = [0.95, 0.5, 0, 0.5], [0.95, 0.7, 0, 0.5], [0.95, 0.95, 0, 0.5], [1, 0.95, 0, 0.5]). Preliminary analysis showed that the sample means and variances of the variables [*T, M, Y*] were within 0.013 bias across replications for all simulation conditions. Similarly, the bias of the correlation coefficient estimates [*rTX, rTM, rMY, rTY*] in each simulation condition was also small, within 0.013. We report these statistics across all simulation conditions in Supplementary Material S3. Significance tests using the Baron and Kenny (1986) method and bootstrapping showed small differences in detection rates, within 0.020 across all simulation conditions; therefore, we report only the bootstrap results.

### Case 1: Successful experimental manipulation and manipulation check variable

**Fig. 3:**
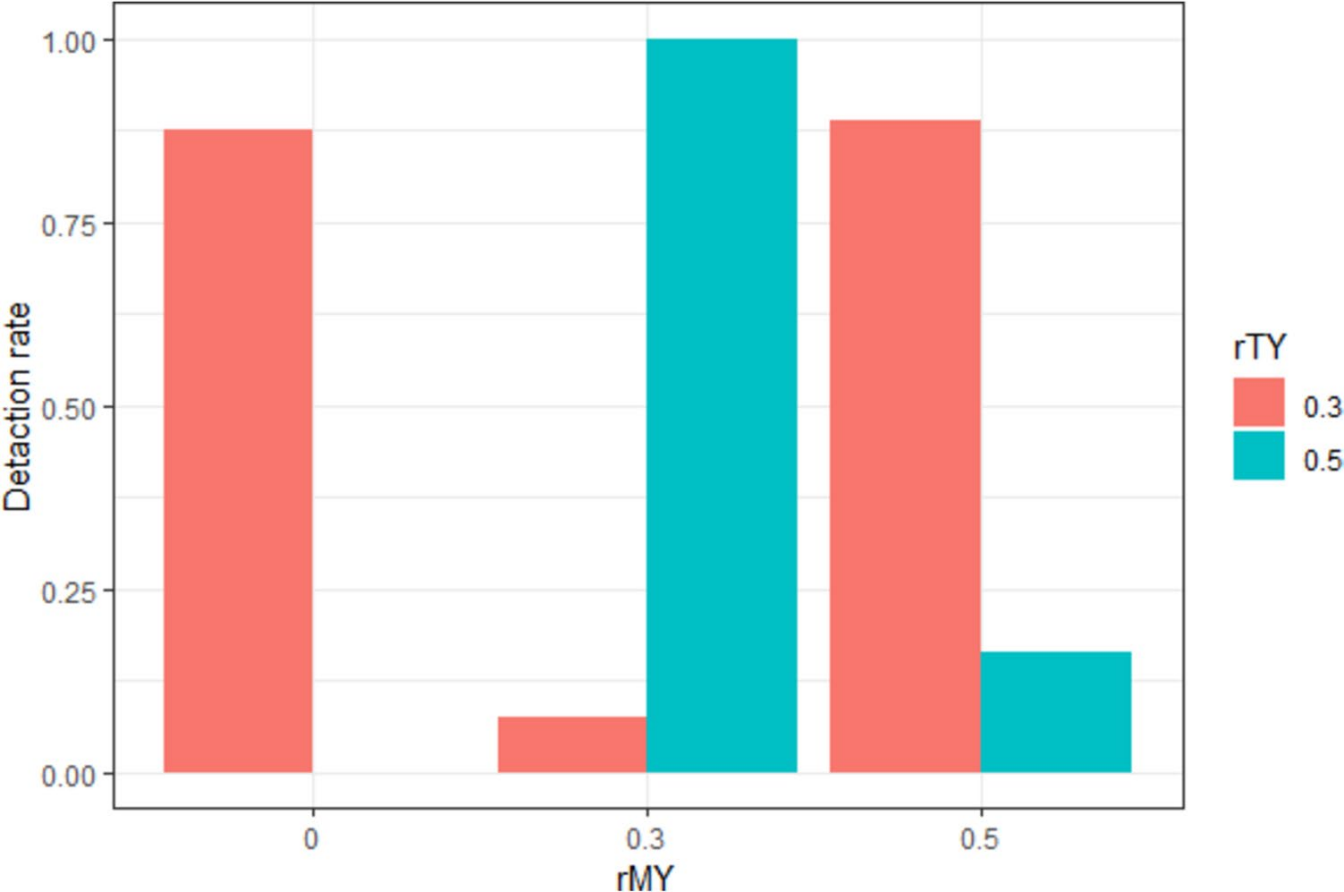
Detection rate of the indirect effects under the simulation conditions in Case 1

Figure 3 shows the mediation effect detection rate as a function of *rMY* and *rTY* when the experimental manipulation is perfect (*rTX* = 1) and the manipulation check variable has the smallest measurement error (*rTM* = 0.95). The rate did not vary systematically with *rMY* and *rTY*. Consistent with the prediction that mediation effect detection rates would not be systematic under conditions of high multicollinearity, we observed both high detection rates above 0.8 and low detection rates around 0.1 when the experimental manipulation and the MC were successful.

Table 1 presents the detection rate, mean, and standard deviation for the indirect effect (*ab*) and the variance inflation factor (VIF) for each simulation condition. The conditions in Case 1 had high multicollinearity with a VIF of greater than 10. The mediation detection rate was affected by the magnitude of the indirect effect. For conditions in which *rTY* and *rMY* had the same parameter, the magnitude of the indirect effect was less than 0.246; therefore, the detection rate of the indirect effect was less than or equal to 0.163. In contrast, for conditions in which *rTY* and *rMY* were not equal, the absolute value of the indirect effect size was greater than 1.7, and an indirect effect was detected 100% of the time. Because all the variables were standardized, effect sizes greater than 1.0 are extreme. Conditions in which *rMY* was smaller than *rTY* ([*rMY,rTY*] = [0.0,0.3], [0.3,0.5]) showed a negative indirect effect. The fact that *rMY* is smaller than *rTY* in the mediation analysis is not relevant to the interpretation that the effect of the independent variable is due to the manipulation check variable. Rather, it suggests that the manipulation check variable is not an effective mediator when *rMY* < *rTY* in Case 1 due to the inconsistent mediation effect.

**Table 1:** Statistics for the indirect effect (*ab*) and VIF under the simulation conditions in Case 1

Simulation conditions				Statistics				
*rTX*	*rTM*	*rMY*	*rTY*	Detection rate	*M*		*SD*	VIF
1.0	0.95	0	0.3	1.000		− 2.775	0.122	10.645
1.0	0.95	0.3	0.3	0.088		0.136	0.295	10.603
1.0	0.95	0.3	0.5	1.000		− 1.714	0.204	10.640
1.0	0.95	0.5	0.3	1.000		2.096	0.215	10.656
1.0	0.95	0.5	0.5	0.163		0.246	0.264	10.612

*M,* mean; *SD,* standard deviation

### Case 2: Successful experimental manipulation and erroneous manipulation check variable

**Fig. 4:**
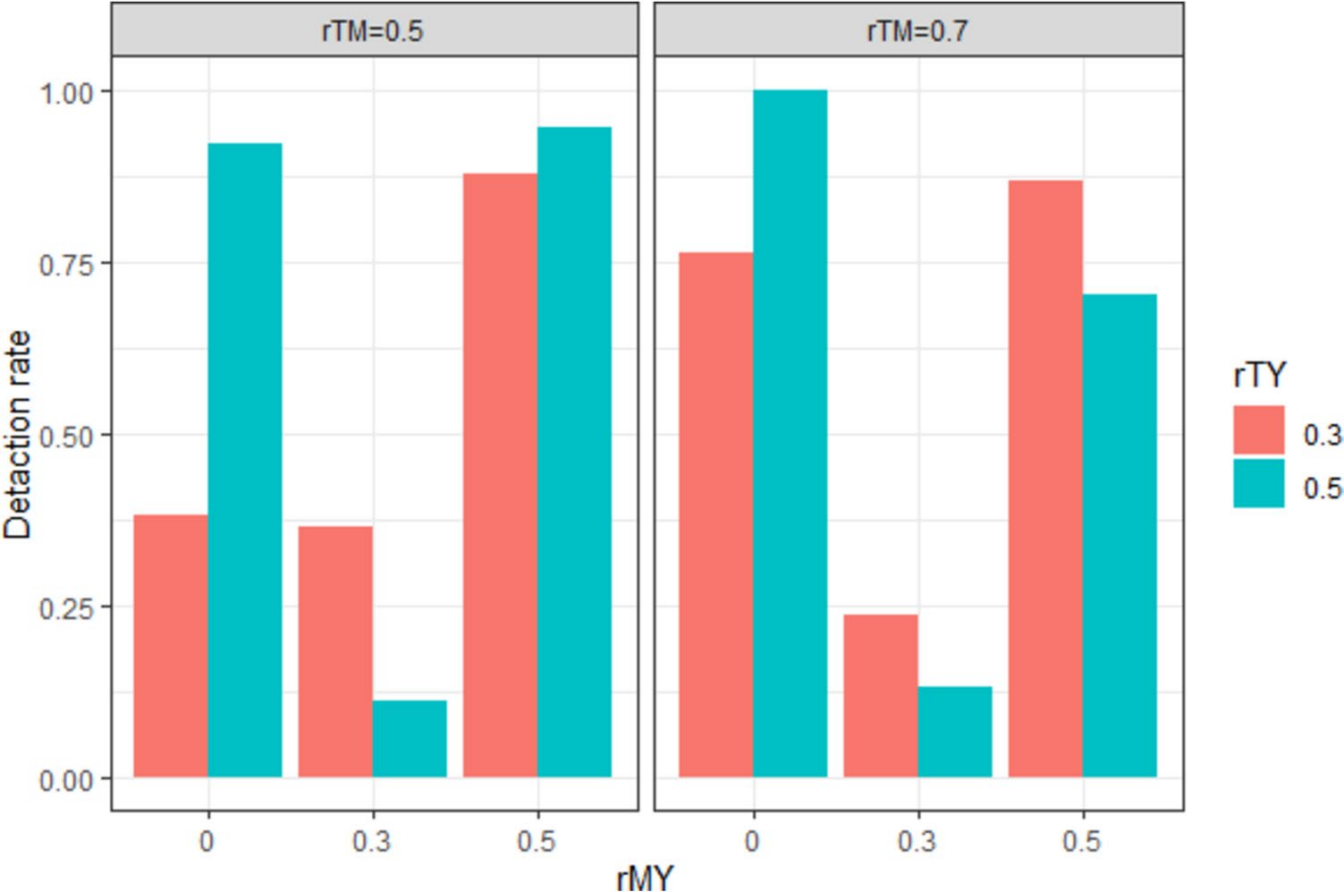
Detection rate of indirect effects under the simulation conditions in Case 2

Figure 4 shows the detection rate of the mediation effect as a function of *rMY* and *rTY* at the levels where the experimental manipulation is successful (*rTX* = 1) but the manipulation check variable contains measurement error (*rTM* = 0.5 and 0.7). For the conditions in Case 2, the detection rate did not change systematically with *rMY* and *rTY*. Notably, the detection rate changed in a U-shape as *rMY* changed. To find the reason for the U-shaped pattern, we investigated the statistics of the indirect effects in the Case 2 conditions (Table 2). The high detection rate of indirect effects at *rMY* = 0 was due to an inconsistent mediation situation. In these simulation conditions, the indirect effects were all negative (− 0.484 to − 0.100). In contrast, at *rMY* = 0.5, the signs of the indirect effects were all positive (0.169 to 0.402), and at *rMY* = 0.3, the magnitude of the indirect effects was close to zero (− 0.069 to 0.126). This finding raises the possibility that the detection rate of indirect effects may not accurately test the hypothesis the researcher is trying to test, that is, whether the effect of the experimental condition is caused by psychological attributes.

**Table 2:** Statistics for indirect effect (*ab*) under the simulation conditions in Case 2

Simulation conditions				Statistics		
*rTX*	*rTM*	*rMY*	*rTY*	Detection rate	*M*	SD
1	0.5	0	0.3	0.442	− 0.100	0.058
1	0.5	0	0.5	0.921	− 0.165	0.058
1	0.5	0.3	0.3	0.427	0.101	0.059
1	0.5	0.3	0.5	0.111	0.033	0.051
1	0.5	0.5	0.3	0.995	0.236	0.066
1	0.5	0.5	0.5	0.946	0.169	0.058
1	0.7	0	0.3	0.883	− 0.289	0.094
1	0.7	0	0.5	1.000	− 0.484	0.087
1	0.7	0.3	0.3	0.269	0.126	0.094
1	0.7	0.3	0.5	0.130	− 0.069	0.087
1	0.7	0.5	0.3	0.990	0.402	0.095
1	0.7	0.5	0.5	0.703	0.208	0.086

*M,* mean; *SD,* standard deviation

Additionally, the *rMY* moderated the relationship between the true effect size (*rTY*) and detection rate. For *rMY* = 0, the detection rate of the indirect effect was higher when *rTY* = 0.5 than when *rTY* = 0.3; however, the opposite was true for *rMY* = 0.3. These results suggest that using mediation analysis when measurement error is present in the manipulation check variable may be more likely to lead to spurious findings of causality when the true effect size is large.

### Case 3: Incomplete experimental manipulation and successful manipulation check variable

**Fig. 5:**
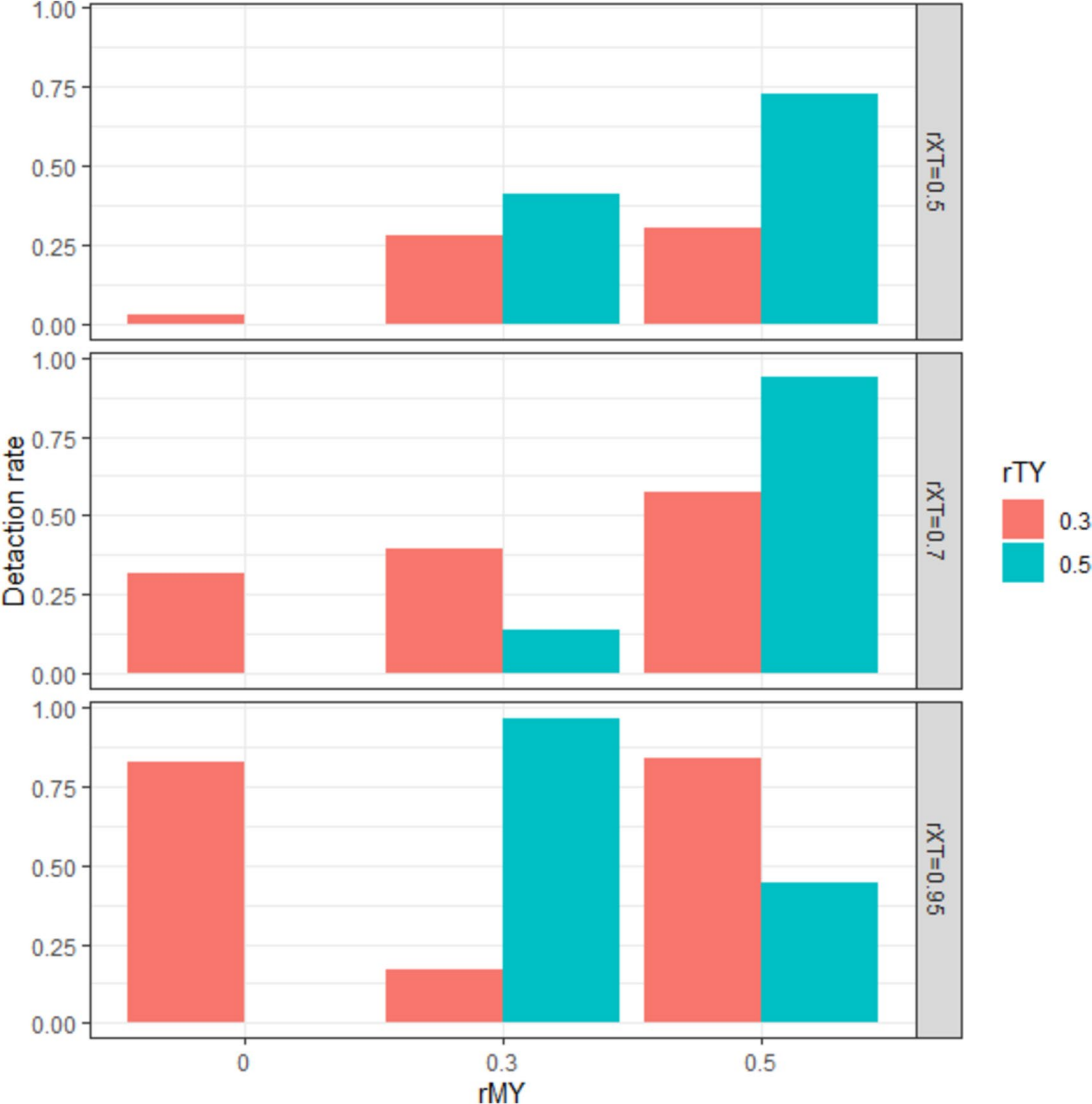
Detection rate of indirect effects under the simulation conditions in Case 3

Figure 5 shows the mediation effect detection rate as a function of the levels of *rMY* and *rTY,* where the manipulation intensity is imperfect (*rTX* < 1), while the manipulation check variable is highly correlated with the psychological attribute (*rTM* = 0.95). Under the conditions of Case 3, it was difficult to find a systematic change in the mediation effect detection rate based on the simulation conditions. However, at the level of *rTX* = 0.5, where the manipulation intensity is small (and therefore, the multicollinearity of *X* and *M* was small), the detection rate of the indirect effect increased as *rMY* increased. This result is consistent with the prediction that the detection rate of indirect effects changes systematically when the multicollinearity of *X* and *M* is small.

### Case 4: Incomplete experimental manipulation and erroneous manipulation check variable

**Fig. 6:**
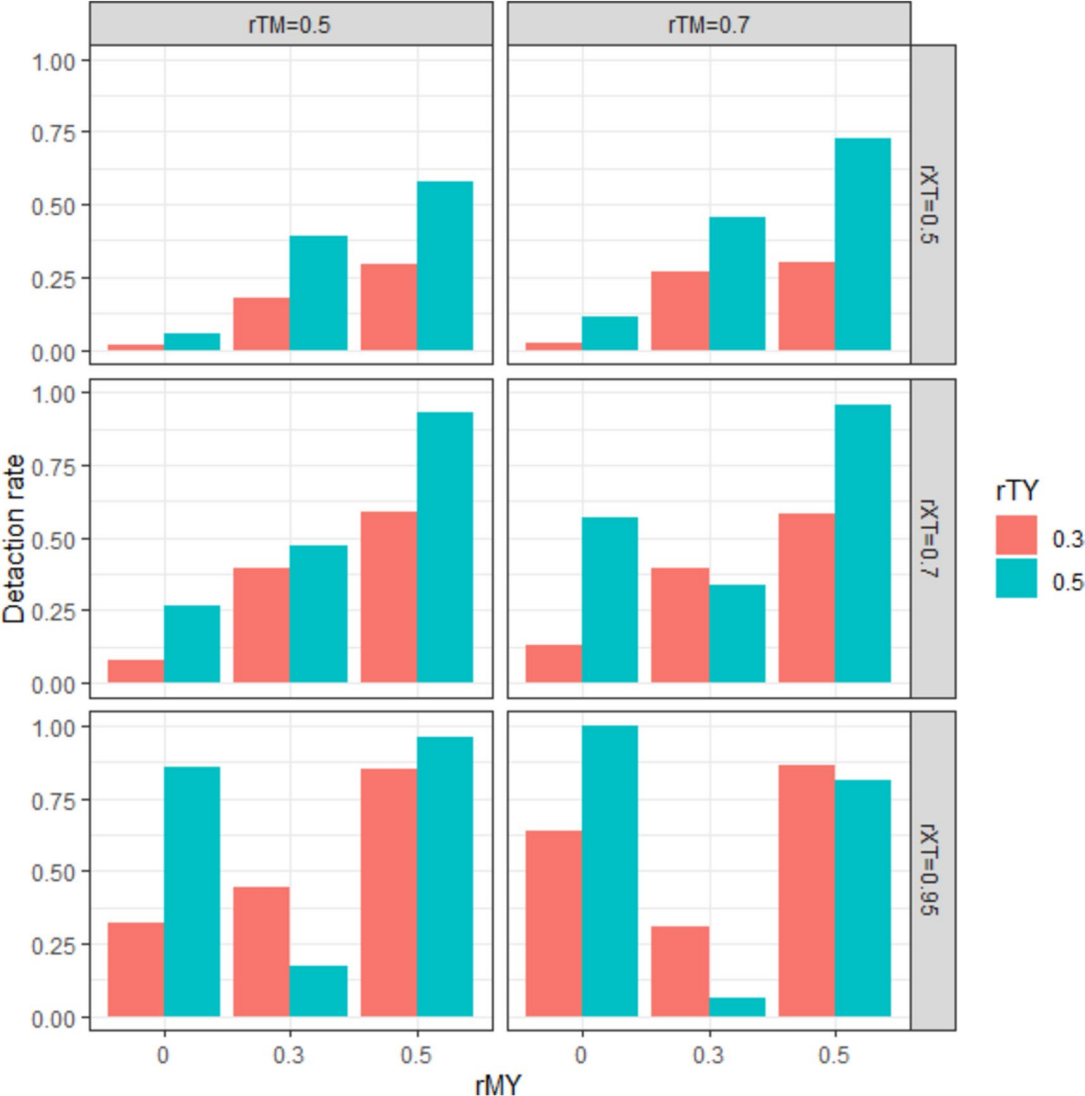
Detection rate of indirect effects under the simulation conditions in Case 4

Figure 6 shows the detection rate of the mediation effect as a function of *rMY* and *rTY* at levels where *rTX* < 1 and *rTM* = 0.5, 0.7. The detection rate of mediation effects increased linearly with increasing *rMY* at low levels of multicollinearity: [*rTX, rTM*] = [0.5, 0.5], [0.5, 0.7], [0.7, 0.5]. However, in conditions where both *rTX* and *rTM* were above 0.7, the pattern of the mediation effect detection rate by experimental condition was similar to Case 2 (i.e., the mediation effect detection rate was higher at the *rMY* = 0 level than at the *rMY* = 0.3 level), and this trend was more pronounced at the *rTY* = 0.5 level. We can infer that this result is due to the negative sign and larger absolute value of the indirect effect at the *rMY* = 0 level, as in Case 2.

## Discussion

The simulation study investigated the results of the TMC procedure by manipulating the correlation coefficients of the variables involved in the EMPs. The true relationship between *T* and *Y* existed in all simulation conditions of this study; however, the test results concerning a causal inference varied dramatically depending on the simulation condition.

The findings can be summarized in three ways. First, the detection rate of indirect effects in the TMC procedure was inconsistent. Not only did the detection rate range from 0 to 1 across the simulation conditions, but the results also could not be explained by the effect of only one or two conditions. Additionally, some mediation situations had a negative indirect effect.

Second, multicollinearity between *X* and *M* severely distorted the mediation analysis results (e.g., Cases 1 and 2). The multicollinearity was larger when the manipulation intensity was successful and the measurement error of the manipulation check variable was small. Why does the multicollinearity of *X* and *M* distort the TMC results? The mediation analysis proposed by Lench et al. (2014) evaluates *M*’s contribution to the effect of *X* on *Y*. Therefore, the indirect effect is the degree to which *M* is “partialing out” the total effect of *X* on *Y*, and a significant indirect effect indicates that, after controlling for *M*, the unique explanation of *X* on *Y* significantly decreases. Furthermore, a negative indirect effect or suppression effect occurs when the partial correlation between *M* and *Y* after controlling for *X* is small while the simple correlation between *X* and *M* is large. However, if *X* and *M* are highly correlated, they will have a greater impact on the mediation analysis, even if the amount of unique explanation for *Y* in each of *X* and *M* is very small. In this case, the detection of a mediation effect does not provide meaningful information to the researcher. Relying on significance alone can also lead to misjudging the suppression effect as a mediation effect. We recommend that researchers employing mediation models distinguish and explicitly hypothesize the role of a modeled variable as a mediator or suppressor.

Third, when individual differences in psychological attributes remain within experimental conditions (e.g., *rTX* = 0.5 in Cases 3 and 4), TMC can be used to check the validity of manipulation checks. When *rMY* = 0, the relationship between the independent and dependent variables is not due to the psychological attributes manipulated by the researcher, as shown in Fig. 2c. The simulation results showed that the detection rate of indirect effects in these conditions was close to zero, and the detection rate increased as *rMY* increased. These results are consistent with those of Lench et al. (2014). Furthermore, as the size of the measurement error became smaller (i.e., as *rTM* increased), the detection rate of indirect effects became larger. This result suggests that the higher the reliability of the manipulation check, the higher the validity of the TMC procedure.

As a simulation study, this study has limitations that do not reflect all experimental situations in the real world. First, the levels of the simulation conditions set in this study did not reflect real-world data. Second, all variables in this study were assumed to be normally distributed with equal variance across groups, and all relationships between variables were linear. These assumptions are oversimplified compared to the relationships between variables found in empirical studies. Third, complex issues such as missing data and double mediation were not considered.

Based on the results of this study and previous research, we suggest the following practices for empirical researchers conducting experiments that manipulate psychological attributes. First, we strongly recommend that manipulation checks be conducted separately from the main experiment (Aronson & Carlsmith, 1968; Festinger, 1953; Hauser et al., 2018; Westlund & Stuart, 2017). If the validity of the manipulation is called into question in the main experiment, there is no way to improve the validity. TMC is not a way to make causal inferences stronger, but rather an auxiliary means to explain the relationship when *X* → *Y* is significant (Lench et al., 2014). Therefore, even if TMC is used, checking the validity of the manipulation in a pilot experiment is appropriate.

Second, we recommend finding the correlation coefficient between the observed variables before conducting TMC and checking whether the conditions are such that the procedure can show valid results. If the conditions are not met, not using the TMC procedure may be more valid. For example, if the correlation coefficient between the observed independent variable and the manipulation check is too high, the TMC results are likely to be biased by multicollinearity. If the correlation coefficient between the manipulation check and the dependent variables is too low, the TMC results are also unreliable.

Third, speculating about the relationship between the observed and latent variables is necessary. The effectiveness of a manipulation can vary depending on the context of the study. For example, in studies where experimental manipulations can qualitatively change psychological attributes, such as psychological distance (intimate vs. stranger) or social membership (ingroup vs. outgroup), the between-group differences in psychological traits may be large enough to outweigh the within-group variability. In other cases, ethical considerations limit the strength of the psychological trait that the researcher can manipulate. In the latter case, the distribution of the psychological attribute has a large overlap between groups. The TMC procedure may be useful in the latter case.

Fourth, it is recommended to pay attention to the reliability and validity of the manipulation check variables. The reliability of the manipulation check variable also indicates the relationship between the observed and latent variables. If multicollinearity is not an issue, the higher the reliability of the instrument, the higher the validity of the manipulation check procedure. It is important to use an instrument with known high reliability or to check the reliability coefficients of the measured manipulation check variables. It is also necessary to make sure that *X* is changing psychological attributes other than *T* in order to ensure the internal validity of the study. One way to do this is to measure other potential confounding variables as well as the manipulation check variables.

Finally, when conducting TMC procedures, it is important to consider not only the significance of the indirect effects, but also the sign of the indirect effects. Relying solely on the significance of the indirect effect to determine the validity of the manipulation test does not rule out alternative explanations, such as inconsistent mediation effects.

## Data Availability

Not applicable.

## Code availability

The R code for data generation and analysis in this study is provided in Supplementary Material S1 and available in the authors’ OSF repository (https://osf.io/hwkq3/).
